# Pulmonary Manifestations of Primary Immunodeficiency Disorders in Children

**DOI:** 10.3389/fped.2014.00077

**Published:** 2014-07-25

**Authors:** Milos Jesenak, Peter Banovcin, Barbora Jesenakova, Eva Babusikova

**Affiliations:** ^1^Center for Diagnosis and Treatment of Primary Immunodeficiencies, Department of Pediatrics, Jessenius Faculty of Medicine, Comenius University in Bratislava, Martin, Slovakia; ^2^Department of Medical Biochemistry, Jessenius Faculty of Medicine, Comenius University in Bratislava, Martin, Slovakia

**Keywords:** infectious complications, inheritance, immune system dysregulation, interstitial lung diseases, respiratory tract, non-infectious complications, primary immunodeficiencies

## Abstract

Primary immunodeficiencies (PIDs) are inherited disorders in which one or several components of immune system are decreased, missing, or of non-appropriate function. These diseases affect the development, function, or morphology of the immune system. The group of PID comprises more than 200 different disorders and syndromes and the number of newly recognized and revealed deficiencies is still increasing. Their clinical presentation and complications depend on the type of defects and there is a great variability in the relationship between genotypes and phenotypes. A variation of clinical presentation across various age categories is also presented and children could widely differ from adult patients with PID. Respiratory symptoms and complications present a significant cause of morbidity and also mortality among patients suffering from different forms of PIDs and they are observed both in children and adults. They can affect primarily either upper airways (e.g., sinusitis and otitis media) or lower respiratory tract [e.g., pneumonia, bronchitis, bronchiectasis, and interstitial lung diseases (ILDs)]. The complications from lower respiratory tract are usually considered to be more important and also more specific for PIDs and they determinate patients’ prognosis. The spectrum of the causal pathogens usually demonstrates typical pattern characteristic for each PID category. The respiratory signs of PIDs can be divided into infectious (upper and lower respiratory tract infections and complications) and non-infectious (ILDs, bronchial abnormalities – especially bronchiectasis, malignancies, and benign lymphoproliferation). Early diagnosis and appropriate therapy can prevent or at least slow down the development and course of respiratory complications of PIDs.

## Introduction

**Figure 1:**
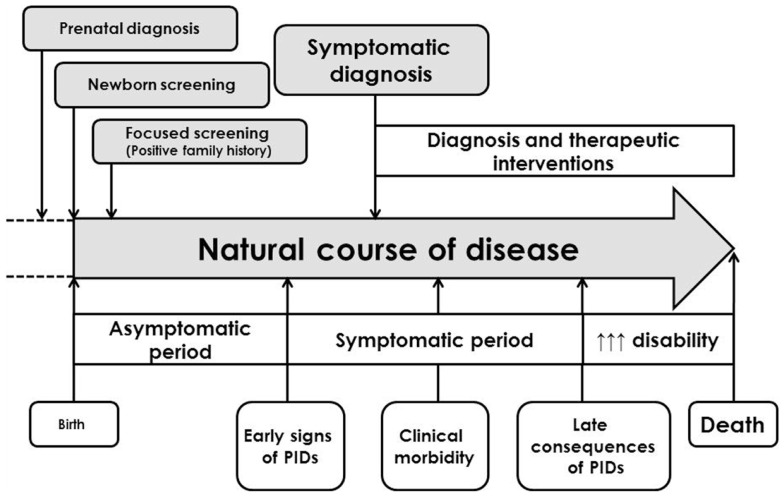
**General clinical course of primary immunodeficiencies and different approaches in establishment of the diagnosis [adapted and modified from Ref. (4)]**.

Science of primary immunodeficiencies (PIDs) represents a fascinating and rapidly developing part of modern medicine and clinical immunology. PIDs are inherited disorders of immune system in which one or several immune components are decreased, missing, or of non-appropriate function. These diseases affect the development, function, or morphology of the immune system (1). Since the first official scientific publication of the PID case in 1952 (2), more than 200 other diseases were described and characterized. The heterogeneity of the PIDs, the variability of their clinical manifestations, inconsistence in the relationship between genotype and phenotype and involvement of whatever organ or tissue supports the interdisciplinary character of these diseases, which requires multidisciplinary approach in their management. Hand-in-hand, continual education of health professionals and laics as well is urgently needed. There are on-going efforts of the education of laic and medical community, which is aimed on the increase of public awareness of the PID. The early diagnosis is usually associated with better prognosis and sooner appropriate therapeutic strategies. From the symptomatic diagnosis that was based on the detection of various immune system defects after the onset of clinical symptoms and complications, we shifted toward complicated algorithms consisting from different immunological tests accompanied by molecular-genetic analysis in the selected cases (Figure 1). The biggest challenge of current immunology is to establish the diagnosis of PID even before the onset of the clinical symptoms, just to start the right therapy as soon as possible and to prevent the possible complications and consequences of non-treated or non-appropriately treated disease. Several countries have recently introduced into the praxis the pilot programs of neonatal screening for selected immunodeficiencies [especially for severe combined immunodeficiency (SCID) diseases, but also for ataxia telangiectasia and some severe humoral immunodeficiencies]. On the other hand, for the most severe diseases, the prenatal diagnosis in cases of positive family history for particular PID is fully recommended (3).

The group of PIDs is getting more and more heterogeneous and the number of newly recognized and revealed deficiencies is increasing year by year. The majority of PIDs is monogenic, but in some cases, the polygenic basic is suspected. There are still many PIDs with unknown or not-fully described and characterized genetic background [e.g., common variable immunodeficiency (CVID) or selective deficiency of IgA]. PIDs are usually rare diseases with overall prevalence of 1:10,000 live births. However, some of the PIDs can be found quite frequently (e.g., selective deficiency of IgA, deficiencies of IgG subclasses, deficiency of mannose-binding lectin, etc.) (5). The most frequent immunodeficiencies yield usually mild course. Some of the frequent immunodeficiencies can be even asymptomatic during the lifetime and their clinical significant of questionable and discussed. In case of the combination of different “mild” defects in immunity, the clinical manifestation becomes more possible and evident.

The classification of PIDs underwent a long way and was elaborated regularly. The understanding of the particular diseases allowed the creation of the new categories and groups of related diseases of immune system. According to the current knowledge, the immunodeficiencies are classified into the eight major categories based on the primarily involved immune component and associated symptoms and signs (6):

1. predominantly humoral (antibody) deficiencies,
2. combined T-cell and B-cell immunodeficiencies,
3. other well defined immunodeficiency syndromes,
4. congenital defects of number and/or function of phagocytes,
5. complement deficiencies,
6. defects of immune dysregulation,
7. autoinflammatory disorders,
8. defects in innate immunity.

Among all the immunodeficiencies, antibody deficiencies are the most frequent and comprise approximately 70–75% of all PIDs. These patients are typically characterized by different respiratory symptoms and complications due to the inherited immune defect. In children, respiratory symptoms are typical initial presentation of various PIDs. However, also the other groups and classes of PIDs can be associated with significant respiratory morbidity and manifestations (Table 1). Through two simple widely available test – serum immunoglobulin concentration (IgG, IgA, IgM, and ±IgE) and differential leukocyte cell count – the majority of the PID can be detected and revealed. Therefore, these two simplex tests can be in general recommended as screening tools for PIDs in primary care.

**Table 1:** **Respiratory presentations and complications of primary immunodeficiencies [adapted according to Bierry et al. (7) and Touw et al. (8)]**.

Non-infectious complications	Infectious complications	Chronic lung disease	Chronic inflammatory diseases	Benign lymphoproliferative disease	Malign neoplasma
**RESPIRATORY COMPLICATIONS OF PRIMARY IMMUNODEFICIENCIES**					
Bronchial abnormalities (bronchiectasis, bronchial wall thickening, atelectasis, mucus plugs, emphysema, bullae, pneumatocoele)	Otitis	Fibrosis	Granulomas	Parenchymal lymphoid hyperplasia	Solid organ tumors (leiomyoma, adenocarcinoma)
Lung parenchyma abnormalities (nodules, cavity)	Rhino/sinusitis	Pulmonary hypertension	Interstitial lung disease	Reactive follicular hyperplasia	Lymphomas
Ventilation abnormalities (obstructive, restrictive, combined)	Bronchitis	Cor pulmonale		Mediastinal lymphadenopathy	Thymic tumors
Laryngeal angioedema	Pneumonia	Respiratory failure			Lung metastasis
	Empyema	Allergies			
	Lung abscess				

## Respiratory Manifestations of Primary Immunodeficiencies

Respiratory symptoms and complications present a significant cause of morbidity and also mortality among patients suffering from different forms of PIDs (Table 2). Computed tomography (CT) in combination with other imaging techniques, clinical tests, and laboratory examinations play an important role in detecting, characterizing, and quantifying the extent and kind of lung damage (9). Another important role of the imaging techniques lies also in the evaluation of the lung pathology progression. The screening for lung complications should be performed regularly, especially in patients with antibody deficiencies. Based on the expected symptoms and complications, the so-called *warning signs for primary immunodeficiencies* were elaborated (Tables 3 and 4). Respiratory complications, especially infectious can be expressed very soon in the early life (Table 5). The non-infectious complications and manifestations usually appear during the course of PIDs in the adolescent or adult age. Among all age categories, respiratory symptoms present an important marker pointing the attention toward PIDs, although it should be assumed that the sensitivity of particular “warning” signs differs (10). The most relevant are these signs:

- positive family history for PIDs,
- more than 2-months antibiotic therapy for PIDs with the dysfunction of neutrophils,
- failure to thrive ± chronic diarrhea for T-cellular immunodeficiencies.

**Table 2:** **The most important immunodeficiencies associated with respiratory complications in children**.

Mild immunodeficiencies	Severe immunodeficiencies
Transient hypogammaglobulinemia of infancy	Common variable immunodeficiency
Selective deficiency of IgA	Severe combined immunodeficiencies
Deficiencies of IgG subclasses	Congenital neutropenias
Deficiencies of specific antibodies	X-linked agammaglobulinemia
Deficiency of mannose-binding lectin	Hyper-IgE syndromes
	DNA-repair defects (e.g., Nijmegen breakage syndrome)

**Table 3:** **Warning signs for primary immunodeficiencies in children [adapted according to Arkwright and Gennery (11)]**.

1.	≥4 Ear infections in 12 months
2.	≥2 Serious sinus infections in 12 months
3.	≥2 Pneumonias in 12 months
4.	Recurrent, deep skin, or organ abscesses
5.	Persistent thrush in mouth or fungal infection on skin
6.	≥2 Deep-seated infections (septicemia, osteomyelitis, meningitis, etc.)
7.	≥2 months on antibiotics with little or no effect
8.	Need for intravenous antibiotics to clear infections
9.	Failure to thrive
10.	Positive family history of primary immunodeficiency

*If someone is affected by ≥warning sign, he should be examined for possible immunodeficiency*.

**Table 4:** **Warning signs for primary immunodeficiencies in adults**.

1.	≥4 Infections treated with antibiotics per year (otitis, bronchitis, sinusitis, and pneumonia)
2.	Recurrent infections or infections require long-term antibiotic therapy
3.	≥2 Serious bacterial infections (osteomyelitis, meningitis, septicemia, and cellulitis)
4.	≥2 Pneumonia during last 3 years
5.	Infections caused by atypical bacteria in unusual location
6.	Positive family history of primary immunodeficiency

**Table 5:** **Respiratory manifestations and complications of primary immunodeficiencies in childhood with estimated average frequency**.

	Respiratory manifestation	Frequency
1.	Respiratory infections (rhinosinusitis, otitis media, bronchitis, and pneumonia)	↑ ↑ ↑
2.	Complications and consequences of respiratory infections (bronchiectasis, lung abscesses, empyema, and pneumatocoeles)	↑ ↑
3.	Airways structural abnormalities (bronchial wall thickening and air-trapping)	↑ ↑
4.	Interstitial lung diseases (lymphoid interstitial pneumonia)	↑
5.	Lymphoproliferative diseases (lymphoma, benign lymphoproliferative diseases, and lymphadenopathy)	Rare

In the retrospective study in 64 children from Egypt, the most frequent symptoms were: need for intravenous antibiotic therapy, gastrointestinal symptoms, and recurrent pneumonias within 12 months (12). There are also some other potentially alarming signs and complications for PIDs:

- autoimmune disease of unknown etiology,
- opportunistic infections,
- complications after the vaccination with live attenuated vaccines (especially after BCG vaccination against tuberculosis),
- chronic graft-versus-host diseases (feto-maternal engraftment),
- systemic atypical mycobacteriosis,
- persistent and recalcitrant dermatitis in infants,
- delayed umbilical cord separation.

**Figure 2:**
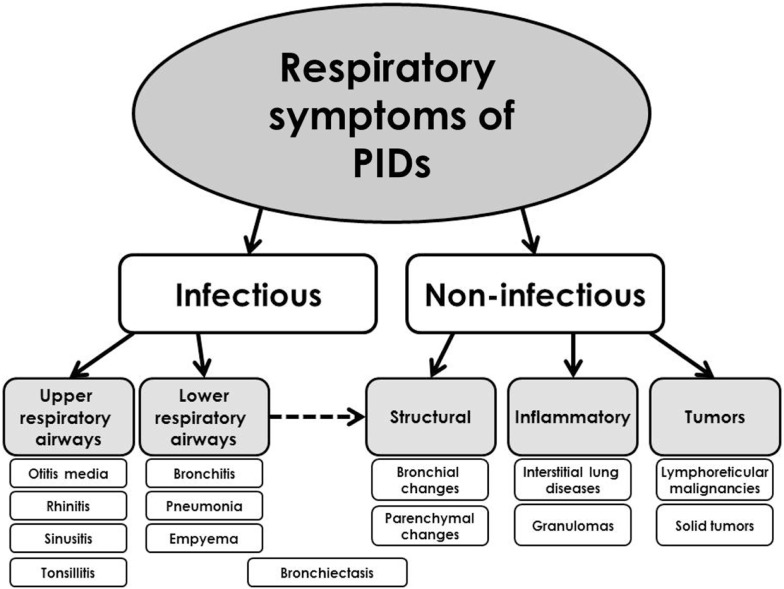
**Simplified classification of the respiratory presentations of primary immunodeficiencies**.

The respiratory symptoms and complications of PIDs can affect primarily either *upper airways* (e.g., sinusitis and otitis media) or *lower respiratory tract* [e.g., pneumonia, bronchiectasis, and interstitial lung diseases (ILDs)]. The complications from lower respiratory tract are usually considered to be more important and also more specific for PIDs and they determinate patients’ prognosis. The respiratory signs of PIDs can be divided into infectious and non-infectious (Figure 2). According to the other classification, they can be divided into several basic categories (9):

1. respiratory tract infections,
2. airways disease,
3. interstitial lung disease,
4. malignant diseases.

### Infectious respiratory manifestations of primary immunodeficiencies

Respiratory infections are universal clinical problem and symptom across the whole childhood. One should discriminate among the child with normal susceptibility to infections, transient increased morbidity without any complications and consequences (so-called physiological respiratory morbidity) and the subjects with increased, severe, complicated respiratory morbidity, which evokes the possible immune defect (13, 14). The respiratory infectious complications show typical spectrum of etiological pathogens according to the immune defect, which can help in the diagnostic algorithm for particular type of PID (Table 6).

**Table 6:** **Etiological agents of respiratory infections according to the PIDs category**.

Antibody deficiencies	T- and B-cell combined immunodeficiencies	Phagocytic immunodeficiencies	Complement immunodeficiencies
**Encapsulated bacteria** (*Streptococcus pneumoniae, Staphylococcus aureus, Haemophilus influenzae*, and *Moraxella catarrhalis*)	**Encapsulated bacteria** (*Streptococcus pneumoniae, Staphylococcus aureus, Haemophilus influenzae*, and *Moraxella catarrhalis*)	**Catalase-positive microorganisms** (*Burkholderia, Pseudomonas, Serratia*, and *Staphylococcus aureus*)	**Encapsulated bacteria** (*Neisseria* sp. and *Streptococcus pneumoniae*)
**Other bacteria** (*Bordetella pertussis*)	**Viruses** (respiratory syncytial virus, adenovirus, parainfluenza 3, paramyxovirus, and cytomegalovirus)	**Fungi** (*Aspergillus, Nocardia*, and *Candida*)	
**Opportunistic bacteria** (*Pseudomonas aeruginosa*)	**Opportunistic bacteria** (*Pseudomonas aeruginosa and Pneumocystis jiroveci*)	**Atypical mycobacteria** (including BCG vaccine strain)	
**Atypical bacteria** (*Mycoplasma* and *Chlamydophilla*)	**Atypical mycobacteria** (including BCG vaccine strain)		
**Fungi** (*Aspergillus* and *Scedosporium*) → hyper-IgE syndrome	**Fungi** (*Aspergillus, Scedosporium, Candida, Histoplasma*, and *Cryptococcus*)		
**Viruses** (rhinovirus, herpes simplex virus, and cytomegalovirus)			

#### Predominantly humoral immunodeficiencies

Predominantly humoral immunodeficiencies represent clinically the most important and largest group of inherited immune defects. Their prevalence widely varied in different populations and geographical settings. The most frequent defects are: selective deficiency of IgA, deficiencies of IgG subclasses, and deficiency of specific antibodies. However, since the frequent diseases usually present clinically only with mild symptoms, the most clinically important disease from this PID category is CVID, which is typically presented by infectious symptoms, especially from respiratory or gastrointestinal tract, but is commonly associated with broad spectrum of different non-infectious complications. The first officially reported PID in the literature was X-linked (Bruton’s) agammaglobulinemia (XLA), which belongs also to this group and yields a broad spectrum of early respiratory complications. There are also some other defects associated with the antibody dysregulation and deficiency, but they were re-classified and now are involved in the other PIDs categories [e.g., Hyper-IgE syndrome (HIES), Hyper-IgM syndrome, etc.].

The most common clinical manifestation of predominant humoral (and combined immunodeficiencies with associated antibody defects) are recurrent and prolonged infections involving the respiratory tract, e.g., rhinosinusitis, otitis media, bronchitis, bronchiectasis, and pneumonias. Respiratory infections in PIDs patients are usually severe, persistent, caused by unusual, atypical, or opportunistic microorganisms and recurrent (acronym S.P.U.R.) in comparison with infections in non-PID patients. The clinical symptoms in humoral deficiencies, typically tend to occur after the first 6 months of life (after the disappearance of maternal IgG), however, recent studies indicate that sino-pulmonary infections may occur earlier (16). According to many studies, clinical history is the most important aspect of suspecting a diagnosis of primary humoral immunodeficiency. Therefore, patients at any age with recurrent upper or lower respiratory infections, where the frequency, severity, course of isolated pathogen is unusual or out of context (e.g., in non-smoking patients), should be investigated for possible humoral or other type immunodeficiency (17). The finding of bronchiectasis in young people should rise the suspicious for possible PIDs. During the examination for the possible primary immunodeficiency, the pathological susceptibility to infections can be demonstrated and identified. These patients have more than eight minor infections per year (until 3 years of age and beyond), severe major infections (pneumonia, sepsis, meningitis, encephalitis, osteomyelitis, septic arthritis, empyema, deep visceral, or skin abscesses) with chronic and relapsing course followed by residues. Typical are the relapses caused by the same pathogen and opportunistic infections are quite frequent (18). The frequency of chest infections including pneumonia varied between 37 and 90% across the studies with antibody deficiencies, and the frequency of recurrent sinus infections was estimated between 19 and 98% (17). In CVID, the recurrent pneumonia, sinusitis, and otitis media appear in the majority of the patients (19). In the recent big international study, which involved more than 2200 CVID patients, pneumonia was the most common clinical sign. Patients with pneumonias had lower trough levels of IgG compared to the patients without recurrent pneumonias (20). The un-controlled and recurrent lower respiratory tract infections in these patients usually lead to the chronic changes expressed by interstitial lung processes or the bronchiectasis development. The diagnosis of primary antibody deficiencies is often delayed, despite the presence of chronic respiratory symptoms (17). Chronic rhinosinusitis but also other infectious complications have a significant negative impact on the life quality of patients with humoral deficiencies (21).

Recurrent pneumonia is one of the most frequent, important, and characteristic sign of primary humoral deficiencies (Tables 2 and 3). The pneumonias in antibody deficiencies are typically caused by *encapsulated bacteria*: *Streptococcus pneumonia, Haemophilus influenzae*, and *Staphylococcus* sp. Since the primary antibody deficiencies are usually associated also with the impaired production of specific antibodies after the vaccinations, the etiological agents could be also the vaccine-preventable infections (e.g., *Bordetella pertussis*). Interestingly, in early disease, *H. influenzae* and *S. pneumoniae* typically cause the exacerbations of respiratory symptoms, while within the progression of lung damage, *Pseudomonas* and *Staphylococcus aureus* become more important and dominant (22). *Mycoplasma* can cause also chronic pneumonitis in X-linked agammaglobulinemia (23). Besides the bacteria, these patients have also the increased susceptibility to *respiratory viral infections*. There are also some differences in the natural course of particular infections, e.g., rhinoviral infections are frequent and prolonged. Severe infections caused by varicella-zoster virus, herpes simplex, and cytomegalovirus have been also reported (9). Some *opportunistic pathogens* (e.g., *Pneumocystis jirovecii, Mycobacterium tuberculosis*) can be also found in patients with antibody deficiencies. In patients with HIES, recurrent staphylococcal infections are very typical. The pneumonias in these patients lead to the bronchiectasis and pneumatocoele formation, which serve as *locus minoris resistentiae* for other infections, especially fungi. Other infectious pathologies, e.g., *lung abscess* and *empyema* have been also described (17, 24).

There are several differences between various forms of severe humoral PIDs regarding the frequency and type of respiratory symptoms and complications. As many as 75–84% of CVID patients have had at least one episode (often multiple episodes) of pneumonia before the diagnosis of PID (25). The risk of chronic lung disease is higher in patients with CVID than in those with XLA, probably due to the longer diagnostic delay in CVID (26, 27). In XLA, less than a third had a chronic lung disease. The IgG trough levels at around 500 mg/l are often inadequate to fully prevent chronic lung disease. The delayed diagnosis is associated with a higher risk of developing chronic lung disease (26, 28). The studies with pediatric patients with CVID have shown that CVID in children presents with the comparable symptoms and complications as in adults, but the later diagnosis significantly negatively influence growth and development. Compared to adults, higher frequency of otitis media can be observed. This is probably the consequence of the anatomic characteristics of upper airways in children (29).

The appropriate therapy using the immunoglobulin substitution and antibiotics usually leads to the significant decline of the frequency and severity of infections with the significant impact on the life quality and prognosis of these patients. However, the application of intravenous immunoglobulins can be on the other side associated rarely with pulmonary complications. Mild wheezing or dyspnea is not uncommon immediate reactions during the application and respiratory symptoms are constant part of systemic allergic reactions, which can also occur in association with this treatment. Serious but very rare pulmonary complications include pulmonary embolism, edema, pleural effusion, and transfusion-related lung injury with fever and hypotension. Despite these reactions, immunoglobulin therapy is highly effective and safe and in patients with reactions to intravenous application a shift to subcutaneous route with excellent safety profile is strongly recommended (30).

#### Combined and other well defined immunodeficiencies

Combined T- and B-cell immunodeficiencies represent usually very severe form of immune defects, which requires soon diagnosis and appropriate treatment. The most important disease from this PIDs category is SCID, which is caused by defects in nearly 20 genes. Combined immunodeficiency is typically associated with several complex syndromic immunodeficiencies, which are now involved in other PIDs categories, e.g., other well defined immunodeficiencies (e.g., immunodeficiencies with DNA-repair defects).

Patients with combined T- and B-cell immunodeficiencies have predisposition to infections caused by intracellular pathogens. SCID presents an immunological emergency with very soon presentation. A common feature of SCID infants is the lack of thymic shadow on chest X-ray. The respiratory tract is the most common site of infections in SCID and the most frequently involved microorganisms are *P. jirovecii*, cytomegalovirus, adenovirus, parainfluenza virus type 3, and respiratory syncytial virus (26). Another possible marker for SCID is chronic RSV or persistent bronchiolitis. The infections are severe, prolonged, and complicated (31). *Pneumocystis* pneumonia initially cause diffuse interstitial infiltrates, which progress to alveolar infiltrates, which can be focal and asymmetric (26). Although pneumonia caused by *P. jirovecii* is a common presentation feature of SCID, it is rarely recognized. The diagnosis should be estimated through bronchoalveolar lavage or lung biopsy. The diagnosis is suspected due to infiltrates on chest X-ray and presence of respiratory distress, especially in combination with other symptoms of PID such as diarrhea, failure to thrive, or thrush. *Pneumocystis* infection alone in child with SCID does not lessen the chances of successful hematopoietic stem cell transplantation (HSCT), which is the unique causal therapeutic option for these patients (32). Respiratory viruses, particularly paramyxoviruses and adenoviruses are common significant pathogens in these patients with significant worsening effect of BMT outcome. Aggressive treatment (virostatics, immunoglobulins – intravenous, subcutaneous, or nebulized, corticosteroids) may reduce viral replication, lung damage and may improve respiratory functions and outcome. No treatment can probably results in viral clearance without successful T-cell engraftment (33).

Hyper-IgE syndrome (Job’s syndrome) is a complex combined immunodeficiency. Till today, at least three types of HIES were discriminated (autosomal dominant form – caused by mutation in signal transducer and activator of transcription, STAT3; and two autosomal recessive forms – caused by mutation in gene for tyrosine kinase 2, *TYK2* or gene for dedicator of cytokinesis, *DOCK8*). In general, in HIES the lung symptoms are very common and early presentation of the disease. At the beginning, the recurrent sino-pulmonary infections are caused predominantly by *S. aureus*, and less frequently with *S. pneumonia* and *H. influenzae*. Recurrent pneumonias are typical clinical feature for all the three types of HIES. The healing after infections is usually aberrant and the result is the formation of bronchiectasis and pneumatocoeles, which are considered to be the pathogenic marker for autosomal dominant form of HIES (STAT3 mutation) whereas they were not reported in DOCK8 or TYK2 deficiencies. The pneumatocoeles can be occupied by *Aspergillus* or *Scedosporium* and are difficult to manage and treat. Pneumatocoeles are unusual in children infections and their appearance should alert to an unusual diagnosis are the very least (34, 35). Ones the parenchymal lung damage is present the spectrum of pulmonary pathogens shifts toward the spectrum seen in cystic fibrosis – *Pseudomonas aeruginosa* and non-tuberculous mycobacteria. The lung complication in HIES may be due to the impaired Th17 cell differentiation (36). Prior to pyogenic pneumonias, also HIES can be clinically presented by *P. jirovecii* pneumonia, however, in general this complication is rare. *P. jirovecii* can cause pneumonia in patients with HIES both with and without chronic lung diseases (37).

Patients with Hyper-IgM syndromes could have distinct clinical infectious complications based on the type of genetic background and exact type of the syndrome. While autosomal form of HIMS present as typical humoral PID, the X-linked form shows the spectrum of pathogens similar to the patients with combined immunodeficiencies. The underlying infectious cause of the pneumonias includes encapsulated bacteria, CMV, histoplasmosis, and *P. jirovecii*. Fungal pneumonias caused by *Candida, Cryptococcus*, and *Histoplasma* can be also found (38).

Pneumonia and chronic lung disease can be observed also in the patients with DNA-repair deficiencies (ataxia telangiectasia, Bloom syndrome, and Nijmegen breakage syndrome). The patients with ataxia telangiectasia are susceptible to recurrent viral and bacterial infections, but have also the increased risk of different malignancies, especially lymphoreticular. The increased sensitivity to ionizing radiation should be taken into account when indicating imaging in patients with DNA-repair defects (31).

#### Phagocytic immunodeficiencies

Phagocytic diseases are caused either by the decrease number and/or dysfunction of phagocytes. This group of PIDs is quite rare, however, in case of organ or deep skin abscesses, chronic dermatitis, persistent fungal infections, or delayed umbilical cord separation, one should think about them. The most important disease from this PIDs category is chronic granulomatous diseases (CGD), which is caused by the inability of the phagocytes to produce reactive oxygen species for intracellular killing of ingested microorganism. Another important group of phagocytic immunodeficiencies is presented by different form of *congenital neutropenia* (e.g., severe congenital neutropenia and cyclic neutropenia).

Patients with phagocytic diseases are at increased risk of recurrent respiratory infections. In CGD, the most important pathogens are *S. aureus, Klebsiella, Aerobacter, Pseudomonas, Aspergillus*, and *Candida*. The infections are difficult to treat, slow to resolve, and commonly recur (31). Also some rare and unusual pulmonary infectious complications have been reported in patients with CDG, e.g., pulmonary botryomycosis (39). In phagocytic diseases, the common pathogens are catalase-positive microorganisms (e.g., *Pseudomonas, Burkholderia*, and *Serratia*) or molds (especially *Aspergillus*) (15, 40). Invasive aspergillosis is the leading cause of mortality and morbidity in chronic granulomatous disease, which reflects the key role of the phagocytes NADPH oxidase in host defense against opportunistic fungi. Lung aspergillosis together with recurrent bacterial pneumonia was observed also in patients with severe congenital neutropenia. Their incidences have been significantly reduced as a result of the therapy with recombinant granulocyte colony-stimulating factor (41). In the early phase of disease, plain radiographs or CT scans may show non-specific, patchy, nodular opacities or segmental or lobar consolidation. Sometimes *Aspergillus* nodules may be very small and not visible on plain radiograph (42). They have a typical appearance on CT scans: halo of ground-glass attenuation representing pulmonary hemorrhage. Later on, the cavitation may develop within time with the “air crescent sign” in radiograph (26).

#### Complement immunodeficiencies

Complement immunodeficiencies are supposed to be the rarest type of PID; however, they are probably underreported and underdiagnosed. There is an increased risk for *pyogenic infections* with deficiencies of early components of classical pathway (C1–C4). Deficiencies of terminal complement components (C5–C9) are associated with increased susceptibility to *Neisseria* sp. Deficiency of C3 results in serious complications such as *recurrent pneumonia* (*S. pneumoniae*), meningitis, and peritonitis (43). Specific clinical presentation from the respiratory system is associated with hereditary angioedema, which is clinically manifested by recurrent angioedemas in different part of the body. Potentially life-threatening are the angioedemas located to larynx (15). The age of onset, frequency of attacks, and the factors triggering upper airway swelling are variable among different patients. To avoid a fatal outcome of laryngeal swelling, the therapy should be administered as soon as possible. The new available drugs for the treatment of acute attacks significantly improved the prognosis of these patients (44).

### Non-infectious respiratory complications of primary immunodeficiencies

**Figure 3:**
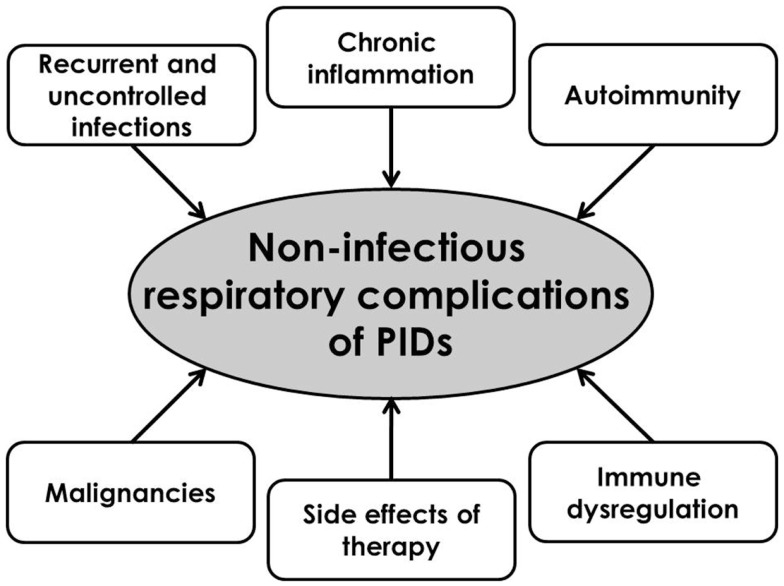
**Pathophysiological substrates of the development of respiratory complications of primary immunodeficiencies**.

Non-infections respiratory complications of PIDs can be the results of recurrent pulmonary infections or are the consequence of the PID itself. The recurrent pyogenic bacterial pulmonary infections lead to the air-trapping, bronchial wall thickening, atelectasis development, and bronchiectasis (9). Final development of chronic respiratory changes is the consequence of inter-play among different factors and mechanisms (Figure 3).

#### Bronchiectasis

Bronchiectasis represents a common consequence of non-controlled chronic infections of lower airways. Among the most frequent causes of bronchiectasis in children, cystic fibrosis, primary ciliary dyskinesia, and immunodeficiencies should be listed (45). The commonest cause of bronchiectasis is *cystic fibrosis*, but neonatal screening reveals these children sooner before this clinical complication. *Primary immunodeficiencies* are among the most important causes for secondary bronchiectasis. Unfortunately, they are diagnosed late in the irreversible phase leading to the end-stage lung disease. On the other hand, their incidence in association with PID is much lower in children compared to adults. Bronchiectasis can be seen also in early-onset CVID in children as one of the main long-term complication in a comparable frequency than in adults (29). The regular screening for this important and mutilating complication is strongly recommended, especially in humoral and T- and B-cell combined immunodeficiencies. The bronchiectasis occurs earlier in *X-linked agammaglobulinemia* compared to *CVID*, probably due to the sooner onset of pulmonary infections in the first one. The bronchiectasis is usually in the lower or middle lobes. It seems that the majority of the hypogammaglobulinemic patients suffer from the mild type of bronchiectasis (46). Bronchiectasis and bronchial wall thickening occurs in 17–76% of antibody deficiency patients and are well recognized complication in these patients (17). Up to 73% of CVID patients develop chronic structural pulmonary complications, of which bronchiectasis and bronchial wall thickening are most frequently detected (8, 47). In *milder form of humoral PIDs* (e.g., IgA deficiency, IgG subclass deficiencies), the incidence of bronchiectasis is much lower than in XLA or CVID. Recent big study with CVID patients showed that bronchiectasis was not associated with any other complications (except for lobectomy). A possible explanation may be that bronchiectasis is the consequence of recurrent and un-controlled infections, whereas other non-infectious complications are the result of immune dysregulation (20).

Regarding the clinical characteristics of bronchiectasis, they are in general cylindrical, bilateral, and diffuse. It is most commonly found in the middle or lower lobes, and less frequently in the upper lobes (7). In the majority of the cases, tubular (cylindrical) bronchiectasis was observed, whereas varicose and cystic type is less common (48). Pathological bronchial findings are mostly observed in the proximal bronchi; meanwhile the involvement of distal bronchi is less common. Chest high-resolution computed tomography (HRCT) should be considered in all patients with chronic chest symptoms to monitor the disease progression, as the chest X-ray remains an insensitive tool for the diagnosis of early pathology (17). Characteristic findings of bronchiectasis on HRCT include bronchial dilatation, bronchial wall thickening, lack of tapering, and bronchi visible closer than 2 cm to the pleural surface (49). Today, regularly HRCT can be recommended as a screening tool for the lung complication of CVID and other antibody deficiencies.

The presence of bronchiectasis at diagnosis predicts poor prognosis, while early diagnosis and aggressive management predict good outcome (22). The diagnostic delay in patients with bronchiectasis is significantly higher than those without the developed bronchiectasis. Some authors suggested that the bronchiectasis secondary to PID in childhood is not always a progressive condition and there is a potential to slow or prevent disease progression with appropriate treatment (49). A significant correlation was found between severe pneumonia/sepsis and the development of bronchiectasis (50). The severity of developed bronchiectasis significantly correlates with clinical symptoms and impaired life quality (51). The majority of CVID patients with bronchiectasis have mild to severe vitamin D deficiency, which could be therefore recommended to be assessed in these patients with subsequent substitution. Vitamin D deficiency could aggravate the risk and length of respiratory infections in patients with CVID (52). The aggressive antibiotic treatment, physiotherapy, and substitution therapy with immunoglobulins in appropriate dose are the most important preventive strategies. The appropriate treatment (immunoglobulins and antibiotics) may delay the development and also alter the natural course of bronchiectasis. The earlier the immunoglobulin substitution is started, the lower probability of bronchiectasis development and need for invasive surgical interventions can be seen at least in some patients (29, 53). Recent study has showed that bronchiectasis secondary to PID in childhood is not always a progressive condition and appropriate treatment can slow or even prevent disease progression (49). However, several authors reported development of bronchiectasis despite IVIG therapy, probably due to persistent local inflammation and mucous obstruction (54, 55). Anyway, efficient immunoglobulin substitution supported by antibiotics when required seems to promote normal growth and to inhibit the development of disabling lung disease in PID patients (56). In general, there are several options and approaches how to treat both infectious and non-infectious lung complications of PIDs (Table 7).

**Table 7:** **Therapeutic possibilities for the respiratory complications of primary immunodeficiencies**.

1.	Immunoglobulin substitution (intravenously, subcutaneously)
2.	Antibiotics (locally/systemic; prophylactic/therapeutic)
3.	Surgery (otitis, sinusitis, lung pathologies)
4.	Hematopoietic stem cell transplantation
5.	Lung transplantation
6.	Physiotherapy
7.	Immunomodulators (corticosteroids, immunosuppressants, anti-CD20 monoclonal antibodies, growths factors, interferon gamma, etc.)
8.	Other symptomatic therapy (antiphlogistics, mucolytics, bronchodilatators, inhalations, etc.)

#### Interstitial lung diseases and PIDs

Interstitial lung diseases represent one of the most important complications of PIDs and belong to their late-onset symptoms. Their occurrence in childhood is quite rare. The chronic non-infectious complications are the best characterized in patients with CVID, where the lungs are affected with the specific CVID-associated entity called *granulomatous-lymphocytic interstitial lung disease* (GLILD), which currently constitutes an important cause of morbidity and mortality. The lung pathology in sense of ILD includes *lymphocytic interstitial pneumonia*, follicular *bronchiolitis, granulomatous lung disease*, and *organizing pneumonia*. *Follicular bronchiolitis, nodular lymphoid hyperplasia, reactive lymphoid infiltrates*, and *lymphocytic interstitial pneumonia* are all forms of pulmonary lymphoid hyperplasia, which is included within the umbrella term GLILD. GLILD presents the pathologic combination of granulomas and lymphoid hyperplasia (57). GLILD appears to be distinct from the bronchiectasis secondary to recurrent infections and possesses some similarities (but also striking differences) with sarcoidosis (58).

The structural airway disease and ILD in CVID display dissimilar clinical and immunological characteristics, which may influence the diagnosis and follow-up of lung pathology in these patients in the future. The pathogenesis of these two entities differs. While airway structural disease is mainly the cumulative effect of recurrent infections followed by subsequent cicatrization of lung tissue, ILD usually results from immune dysregulation (59). However, some forms of “post-infectious ILD” have been also described (60). Some patients with CVID may display the coincidence of both entities and the prevalence of combined lung disease is higher in adults (59). In a subgroup of CVID patients, the development of granulomatous disease can be observed that may cause ILD in approximately 10% of the cases (so-called sarcoid-like disease). The incidence of pulmonary nodules in a population of CVID patients is high (approximately 20–40%), correlates with splenomegaly and autoimmune phenomena (61, 62). The presence of GLILD is associated with a worse prognosis and increased prevalence of lymphoproliferative disorders (63, 64). Lymphoid interstitial pneumonitis may also be isolated (26). Although the lungs are the most common organ system affected by granulomatous disease in CVID, granulomas can be found also in other organs including skin, liver, spleen, and gastrointestinal tract. The type and severity of lung lesions do not correlate with the type of immunodeficiency or with the severity of the sinusal involvement (65). Whereas the recurrent bacterial pneumonia and bronchial suppuration are the most frequent complications of CVID, reactive interstitial pneumonitis and pulmonary lymphoma are less frequent among these patients (19). ILD appears to be asymptomatic at the initial stage, and therefore, the screening of all (including asymptomatic) CVID patients for lung pathologies should be strongly recommended to facilitate early detection and prevent progression of this disease (59). The etiology of granulomatous diseases is still unknown (66). Many different possible factors inducing the development of GLILD have been evaluated, but one of the most relevant is probably the infection with human herpes virus type 8 (64). Other viruses such as EBV or CMV could be also involved (67). The silent radiological features of GLILD include diffuse interstitial infiltrates on plain radiograph, consolidation, ground-glass opacities, and reticular abnormalities on HRCT (17, 68). GLILD presents with CT findings distinct from the usual airway abnormalities most commonly associated with CVID – pulmonary micronodules, thoracic lymphadenopathy, interlobular septal thickening, and multifocal pulmonary consolidation (69). Common physical, radiographic, and laboratory abnormalities in patients with CVID and granulomatous disease include splenomegaly, hilar, and mediastinal lymphadenopathy with ground glass or nodular opacities in lung parenchyma and reduced number and functions of T-cells.

Patients with *other antibody deficiencies* may also have radiologic and functional evidence of ILD, but their frequency and type are incompletely appreciated. Several histological patterns have been reported including lymphocytic interstitial pneumonitis (LIP), granulomatous interstitial pneumonitis, bronchiolitis obliterans organizing pneumonia (BOOP), and usual interstitial pneumonia (UIP) (60). In patients with antibody deficiencies, especially with repeated respiratory infections ILD is frequent and much more prevalent than expected from general population. The most common immunological abnormality associated with ILD is the deficiency of IgG subclasses. There is no particular histologic or radiologic feature consistently related to a particular immunodeficiency (60). As most of patients with IgA deficiency can produce IgG antibodies, they are less prone to bacterial infections so bronchiectasis is not as common as in XLA or CVID (43). Anyway, there are several reports of chronic lung disease also in selective IgA deficiency, especially when associated with IgG_2_ deficiency (70).

*Pneumatocoeles* are the typical clinical radiological finding in the patients with HIES. They are typical warning characteristic sign for autosomal dominant HIES and they can present the locus for other possible infections, e.g., fungal.

#### Tumors of respiratory tract in primary immunodeficiencies

Malignancy is after infections the second leading cause of death in PIDs. The majority of the tumors is associated with the Epstein–Barr virus (30–60% of cases). In general, the risk of developing malignancies varies from 1 to 25%, but the children with CVID and Wiskott–Aldrich syndrome are at greater risk (31). We can discriminate between *benign lymphoproliferative diseases* (parenchymal lymphoid hyperplasia, reactive follicular hyperplasia) and *malignancies*. The *enlargement of mediastinal lymph node* can occasionally lead to superior vena caval syndrome and CVID was recommended to be included in differential diagnosis of ILDs and hilar lymphadenopathy (71).

Regarding the *solid lung tumors*, there are only few case reports in the literature (*leiomyoma, pulmonary adenocarcinoma*) and this respiratory complication is considered to be in general rare in PID patients (72, 73). Pulmonary comprise due to *metastasis* (origin from, e.g., gastric carcinoma) is more often seen than primary pulmonary malignancy (7). *Thymoma* may occur in combined immunodeficiency and this rare entity is called Good syndrome. Good syndrome occurs in 1–6% of patients with primary humoral immunodeficiencies (74). However, *pulmonary lymphoma* seems to be a serious complication associated with CVID or other PIDs (19, 75). Patients with CVID frequently develop lymphoproliferative disease and the risk for malignant lymphoma is increased by more than 300-fold. *Non-Hodgkin’s lymphoma* (especially high-grade B-cell lymphoma) is more common than Hodgkin’s lymphoma. Approximately 8% patients with CVID develop non-Hodgkin’s lymphoma and in general, <1% of patients with humoral PID develop Hodgkin’s lymphoma (76). There is also a possibility of *benign lymphoproliferative disease* in PID, e.g., parenchymal lymphoid hyperplasia of the lungs (77). Some benign conditions (e.g., lymphocytic interstitial pneumonia) can transform into malignant lymphoma (78).

#### Other respiratory complications of PIDs

In patients with humoral immunodeficiencies, the respiratory allergic or allergy-like symptoms such as *dyspnea, rhinitis*, or *asthma* can be frequently seen (29). Many patients despite the appropriate treatment present with *chronic productive cough*, which is usually the hallmark of chronic sinusitis or bronchitis (79). Despite some similarities in the clinical picture of *primary ciliary dyskinesia* and CVID, these two entities require different therapeutic approach. There is a case report in the literature with both diseases in one patient. Therefore, in a patient with already established diagnosis of chronic lung disease with the deterioration of clinical course, the search for secondary diagnosis should be recommended (80, 81).

Several cases of *primary pulmonary hypertension* were also described in association with primary immunodeficiency (82).

*Primary pulmonary dysgenesis* or even agenesis was discussed to be considered as a part of the spectrum anomalies associated with velocardiofacial syndrome (83).

Recently, a new immunodeficiency syndrome called *pulmonary alveolar proteinosis* has been described. It is characterized by accumulation of pulmonary surfactant, respiratory insufficiency and increased infections due to associated immunodeficiency. This syndrome belongs to the category of phagocytic diseases and is a result of the disturbed surfactant catabolism in alveolar macrophages. It can be either acquired (due to autoantibodies against GM-CSF) or congenital caused by *CSF2RA* mutations. *CSF2RA* encodes the GM-CSF receptor α protein. These patients have increased susceptibility to opportunistic microbial pathogens and increased mortality from un-controlled infections (84). Another new syndrome in two 46XX sisters with *fatal lung fibrosis*, profound combined immunodeficiency and gonadal dysgenesis was also recently described. Comparative genome hybridization and analysis of genes known to be associated with severe immune defects in infancy or gonadal dysgenesis showed no abnormality (85).

The delay in the diagnosis of PID results in the poorer prognosis and considerable morbidity, particularly recurrent pneumonias, resulting in structural lung damage such as bronchiectasis, pulmonary hypertension, and ultimately cor pulmonale (17). The *end-stage lung disease* with the development of *cor pulmonale* and *respiratory insufficiency* has been documented as the most common cause of morbidity in primary humoral immunodeficiencies (25). However, non-infectious lung diseases may occur despite optimal immunoglobulin therapy (28). Therefore, patients developing chronic respiratory symptoms should be managed in a multidisciplinary team, including chest physician, as progression of lung disease may occur despite apparently optimal immunoglobulin therapy (17).

### Diagnosis of respiratory complications of PIDs

Due to the chronic involvement of the respiratory tract (bacterial pneumonia, abscesses, fungal lung disease, and interstitial pneumonia) in PIDs, the changes of lung functions could be logically expected. Although different patterns are typically associated with distinct type of immune defects, there is a substantial overlap in imaging findings (26). Screening examinations, such as lung function testing (LFT) and HRCT of the chest, should be used to evaluate pulmonary status in PID patients (86).

#### Lung function testing in PIDs

Several studies evaluated the changes in pulmonary functions. Normal lung functions can be seen in 45–74% patients. In general, the ventilatory disturbances evaluated by lung function tests can be observed in the majority of the patients with CVID or XLA (87). There are some inter-disease differences, e.g., patients with CVID have more commonly abnormal lung tests compared to the patients with X-linked agammaglobulinemia (88). An obstructive pattern is reported most frequently, although restrictive pulmonary disease is well recognized (17). Small airways involvement leads to ventilation abnormalities and chronic obstructive disease, which are usually irreversible (7). A reduced rate of carbon monoxide uptake and restrictive ventilatory pattern is typically found in CVID patients with ILD (63). A significant correlation between the HRCT score and the predicted values in pulmonary function test has been confirmed in several studies (46), while the others did not find such associations (49, 87). There is a negative correlation between a number of episodes of pneumonia and lung function tests. Recent study has shown that the longitudinal decline of lung functions can be observed in patients with PID. Chronic persistent cough can be used as a marker of the reduction of lung functions (87). One study reported an improvement of lung function tests despite bronchiectasis progression on HRCT in patients after the initiation of immunoglobulin treatment and authors concluded that LFT may serve as a toll for evaluation of the clinical response to immunoglobulin therapy (89). The changes in lung function could be used as an indicator of immunoglobulin treatment efficacy. Based on the significant association between the dose of IVIG and spirometric indices, the use of higher IVIG doses as a protection of lung function decline was recommended (90). Lung functional tests can be used to monitor patients at a more regular basis, although featured by decreased sensitivity for complications (8).

#### Imaging techniques in PIDs

There is an essential role of different imaging techniques, especially CT in the identification and monitoring of pulmonary changes and complications in CVID or some other specific immunodeficiencies. One of the most important diagnostic tools for the detection, quantifying, and characterizing the extent and kind of lung damage is CT. HRCT is the reference standard for the detection of bronchiectasis. The early sign of the possible development of bronchiectasis is the bronchial wall thickening. HRCT appears to be more sensitive for the detection of pneumopathies (with special emphasis on bronchial changes and interstitial lesions) in PID patients than chest X-ray (55, 91). Both HRCT and helical CT proved to be useful tools for monitoring of lung changes associated with PID, especially in symptomatic patients with negative radiographic findings (92).

The patients with severe PID should be regularly examined for the possible respiratory symptoms and complications. In general, LFT can be recommended every 6–12 months with repeated chest X-ray. CT examination should be repeated within 5–10 years after initial presentation of PID unless there is a specific indication or changes in clinical status. If significant abnormalities are found, a repeated CT should be performed every 3–5 years depending on the type of abnormality or clinical stability. If patients are prescribed potentially toxic therapies, e.g., high-dose steroids for GLILD, CT examination should be performed approximately 6 months after the initiation of therapy. Subsequent CT examinations depend on clinical, X-ray, and lung function test changes, but in general should be repeated annually for 1–2 years followed by a decreased frequency is stability is achieved (9, 79).

A special approach should be given to the patients with increased radiation sensitivity (DNA-repair defects, also the part of CVID patients). In these cases, an alternative radiation-free technique alternative to *CT scan* or *chest X-ray* could be *MRI evaluation* of the pulmonary changes and alterations. In recent study, CT and MRI findings were comparable for moderate to severe degrees of bronchial and parenchymal alterations, but weaker concordance between CT and MRI scan was found for lower scores of bronchial abnormalities. Therefore, it should be admitted that CT allows better identification of peripheral airways changes. However, further studies are needed to image quality and involvement of this method into the diagnostic algorithm in PID (93). Actually, low-dose HRCT is still considered to be the standard and the most sensitive method for identification of structural abnormalities and pulmonary complications in CVID at the time of diagnosis and at regular time-points during follow-up, however, with the proper follow-up interval yet to be determined. Annual testing (both spirometry and transfer factor) is useful in the assessment of PID patients, and should not be confined to those with radiological evidence of lung disease (27, 94).

In a study with 58 pediatric patients with PID or cancer (12 with PID) suffering from different respiratory symptoms, the *bronchoscopy and bronchoalveolar lavage* was shown to be clinically useful and established an overall diagnostic rate 94% of patients. Infection rate was 74.2% and infectious agent was isolated in 53% of the cases. In PID patients, different agents were isolated (*Pseudomonas, Enterobacter, E. coli, Acinetobacter, Proteus*, and *Aspergillus*). *P. jirovecii* was identified only in patients without prophylactic therapy with trimethoprim–sulfamethoxazole. Authors stated that these two methods should be considered as an initial diagnostic tool in pediatric PID patients (95).

## Conclusion

Respiratory system is the most common site of the clinical manifestations of different PID both in adults and in children. Infectious and non-infectious respiratory complications determinate the patients’ prognosis. To reduce the morbidity associated with PID the greater awareness of respiratory complications for such patients should be raised. Regular examinations by the appropriate tests should reveal the respiratory pathologies in early stages and should be used also for the monitoring of already existing abnormalities. In general, due to the raising awareness of PIDs the prognosis of these patients gradually improves.

## Conflict of Interest Statement

The authors declare that the research was conducted in the absence of any commercial or financial relationships that could be construed as a potential conflict of interest.
